# Delphi survey on the most promising areas and methods to improve systematic reviews’ production and updating

**DOI:** 10.1186/s13643-023-02223-3

**Published:** 2023-03-28

**Authors:** Mersiha Mahmić-Kaknjo, Vicko Tomić, Moriah E. Ellen, Barbara Nussbaumer-Streit, Raluca Sfetcu, Eduard Baladia, Nicoletta Riva, Angelos P. Kassianos, Ana Marušić

**Affiliations:** 1 Cantonal Hospital Zenica, Crkvice 67, 72000 Zenica, Bosnia and Herzegovina; 2 Sarajevo Medical School, Sarajevo School of Science and Technology, Hrasnička Cesta 3a, 71210 Ilidža, Bosnia and Herzegovina; 3 ST-OPEN, University of Split School of Medicine, Split, Croatia; 4 Department of Research in Biomedicine and Health, Center for Evidence-Based Medicine, University of Split School of Medicine, Split, Croatia; 5 Department of Health Policy and Management, Guilford Glazer Faculty of Business and Management and Faculty of Health Sciences, Ben-Gurion University of the Negev, Beersheba, Israel; 6 Institute of Health Policy Management and Evaluation, Dalla Lana School of Public Health, University of Toronto, Toronto, Canada; 7 Cochrane Austria, Danube University Krems, Krems a.d. Donau, Austria; 8 Department of Psychology, Spiru Haret University, Bucharest, Romania; 9 National School of Public Health, Management and Professional Development Bucharest, Bucharest, Romania; 10 Centro de Análisis de la Evidencia Científica, Academia Española de Nutrición y Dietética, Pamplona, España; 11 Department of Pathology, Faculty of Medicine and Surgery, University of Malta, Msida, Malta; 12 Department of Applied Health Research, University College London, London, UK; 13 Department of Nursing, Cyprus University of Technology, Limassol, Cyprus

**Keywords:** Evidence syntesis, Automation tools, Prioritization

## Abstract

**Background:**

Systematic reviews (SRs) are invaluable evidence syntheses, widely used in biomedicine and other scientific areas. Tremendous resources are being spent on the production and updating of SRs. There is a continuous need to automatize the process and use the workforce and resources to make it faster and more efficient.

**Methods:**

Information gathered by previous EVBRES research was used to construct a questionnaire for round 1 which was partly quantitative, partly qualitative. Fifty five experienced SR authors were invited to participate in a Delphi study (DS) designed to identify the most promising areas and methods to improve the efficient production and updating of SRs. Topic questions focused on which areas of SRs are most time/effort/resource intensive and should be prioritized in further research. Data were analysed using NVivo 12 plus, Microsoft Excel 2013 and SPSS. Thematic analysis findings were used on the topics on which agreement was not reached in round 1 in order to prepare the questionnaire for round 2.

**Results:**

Sixty percent (33/55) of the invited participants completed round 1; 44% (24/55) completed round 2. Participants reported average of 13.3 years of experience in conducting SRs (SD 6.8). More than two thirds of the respondents agreed/strongly agreed the following topics should be prioritized: extracting data, literature searching, screening abstracts, obtaining and screening full texts, updating SRs, finding previous SRs, translating non-English studies, synthesizing data, project management, writing the protocol, constructing the search strategy and critically appraising. Participants have not considered following areas as priority: snowballing, GRADE-ing, writing SR, deduplication, formulating SR question, performing meta-analysis.

**Conclusions:**

Data extraction was prioritized by the majority of participants as an area that needs more research/methods development. Quality of available language translating tools has dramatically increased over the years (Google translate, DeepL). The promising new tool for snowballing emerged (Citation Chaser). Automation cannot substitute human judgement where complex decisions are needed (GRADE-ing).

**Trial registration:**

Study protocol was registered at https://osf.io/bp2hu/.

## Background

Systematic reviews (SRs) are evidence syntheses that serve as valuable support for decision-making in healthcare [1, 2] and social sciences [3, 4]. They can be defined as a summary of studies addressing a specific topic using reproducible analytical methods to collect secondary data and analyse it using systematic and explicit methods to identify, select and critically appraise relevant studies, and to extract and summarize the data [5]. SRs can use to reduce biases and provide evidence to stakeholders such as policymakers, decision-makers, practitioners, researchers, academia, the public, and citizens [6].

Since the 1990s, when organizations like Cochrane, Campbell Collaboration, and the Joanna Briggs Institute (JBI) emerged [1, 7, 8], there has been an increase in both the number of SRs and their utilization to inform policy and practice [9]. Due to the fact that tremendous resources are needed to produce and update SRs, there is a need to automatize the process as much as possible and use the workforce and resources to make it more efficient [10, 11]. This study was conceptualized within the framework of EVidence-Based RESearch (EVBRES) [12], which is a 4-year (2018–2022) EU-funded COST Action CA-17117 with over 40 countries participating globally, aiming to encourage researchers and other stakeholders to use an Evidence-Based Research (EBR) approach while carrying out and supporting clinical research—thus avoiding redundant research. The Action has been extended until 16th April 2023, and as part of the research agenda of a working group (WG3) focusing on improving efficiency in producing and updating systematic reviews [10, 13, 14]. Based on the results of previous activities, we designed a Delphi study (DS) to reach an agreement on prioritising the most promising areas and methods to improve the efficiency of producing and updating SRs. DSs offer a flexible approach to obtaining information regarding how best to allocate professional resources such as knowledge and expertise [15, 16]. This is especially important when the agreement on statement is needed based on the best available evidence. In this study, the expert consensus regarding the most promising areas and methods to improve the efficient production and updating of SRs was pursued.

## Methods

A DS was employed to gain expert insight regarding which areas and methods need to be prioritized to improve efficiency in producing and updating SRs. The DS was conducted exclusively online since face-to-face interaction was neither preferred [15] nor achievable due to Coronavirus disease (COVID-19) pandemic travel restrictions. Recommendations for Conducting and Reporting Delphi Studies (CREDES) [17] were followed throughout the manuscript, excluding parts that were beyond the scope of this project (i.e., external validation and dissemination). Participants were provided with a description of the overall aim of the EVBRES and the specific objective of the DS.

Usually, data from the first round of a DS are solely qualitative [18], but as this survey was informed by a scoping review [10] and a qualitative study [19], the quantitative techniques could be applied as early as in round 1.

Round 1 of the DS was launched on November 15th, 2021, and two reminders were sent 2 weeks apart from each other. Round 1 ended on December 14th, 2021. Round 2 of the DS was launched on April 11th, 2022, and three reminders were sent two weeks apart from each other. The survey ended on May 25th, 2022. Since our study was thoroughly informed by previous EVBRES research [10, 11, 13], agreement was expected to be reached after two rounds. As agreement was reached after round 2, there was no need for round 3, and we closed the DS.

### Participants

As response characteristics of a small expert panel in a well-defined knowledge area are considered reliable in light of augmented sampling [20], 55 experienced authors of SRs were invited using a combination of two non-probabilistic sampling methods, both widely used in Delphi methods [21]: purposive sampling technique followed by criterion sampling in which members of the EVBRES were requested to provide us with personal contacts that met the inclusion criteria for participation. Potential participants were contacted by an email from the EVBRES member and informed what they would be asked to do, how much time they would be expected to contribute, and when, as well as what use would be made of the information they provided. Inclusion criteria were (a) participating in at least 3 SRs as an author; and (b) being a first/last/senior/mentor author in at least one SR. All suggested participants’ names were searched using Google Scholar [22] prior of sending invitations in order to confirm that they satisfied the inclusion criteria.

### Delphi survey design and procedures

#### Survey design

To design the surveys, seven formulation and review sessions were conducted with at least two research team members (1 permanent and 2 alternating research team members). LimeSurvey Professional (LimeSurvey Cloud 3.27.24, LimeSurvey GmbH, Hamburg, Germany) was used to design the online survey.

Round 1 of the DS included sections presented in Table 1.

**Table 1:** Organization scheme of DS questionnaire

Section	Type of data	Variables
Demographic data	Range, not an exact value; in order to minimize a chance to identify participants	Age, gender, years of experience in conducting SRs, number of conducted SRs, number of SRs that they have led, role/s in conducting SRs, and area of employment
Prioritisation	(1) 5-point Likert scale mandatory question regarding whether the step is time/effort/resource-intensive and should be prioritized in future research concerning methods of development and automation Participants were offered fixed statements that a particular step needs to be prioritized in future research and an open-ended field. For each statement, participants had to rate how strongly they agree that the topic is important to include (1—“strongly disagree”, 2—“disagree”, 3—“indifferent”, 4—“agree” and 5—“strongly agree”, “I do not know”) (2) Participants were encouraged to provide arguments for the ratings through open responses	Steps of SR production: (1) project management, (2) formulating the review question, (3) finding previous SRs, (4) writing the protocol, (5) constructing the search strategy, (6) literature searching, (7) de-duplicating, (8) screening abstracts, (9) obtaining full-text, (10) screening full-texts, (11) snowballing-citation chasing/tracking, (12) translating non-English studies into English, (13) extracting data, (14) critically appraising, (15) synthesizing data, (16) GRADE-ing (https://www.gradeworkinggroup.org/)– going from evidence to decision, (17) updating the review to see whether some new studies were published between the search date and the final version of the article, (18) performing a meta-analysis, and (19) writing up the review*
Qualitative section	Open-ended long text field (non-mandatory question) in which participants were invited to freely discuss any issue they find important in that step. Participants were encouraged to provide as many opinions as they felt appropriate	(1) How the methodology of producing SRs can be improved, (2) areas that should be prioritized in future research, and (3) other issues considered important regarding SRs’ production and updating

^*^ Based on work by Tsafnat et al. [23] as well as our team’s previous work [11]

The study was piloted among 10 participants of the EVBRES on June 5th, 2021 and some minor adjustments were made to make the questionnaire more user-friendly and time efficient.

In round 2, areas where agreement was not reached in round 1 were further explored by testing statements with required 5-point Likert answers. This included 2 statements on “snowballing” (“development of better tools is needed” and “automation can be helpful in this area”), 4 statements on “GRADE-ing” (“this step is methodologically well developed”, “potential for automation is low in this step”, “standardization of GRADE assessment may be helpful”, “this step is relatively low resource task”) and 2 statements on “deduplication” (“automation is advanced in this step”, “there is scope for improvement”). Theme “meta-analyzing” was not further explored since participants commented that they considered that it refers to "synthesising data".

### Data analysis

For the prioritisation exercise 5-point Likert-scale based quantitative answers was analysed. When analysing the questions regarding prioritization, it was considered that consensus was reached when two-thirds or more (66.7%) of the participants’ responses reached a certain score range (“agree” and “strongly agree”, or “disagree” and “strongly disagree”). Since our sample was small and skewed interquartile range (IQR) as a measure of variability and median as a measure of central tendency were chosen in order to find out in which range the most of the results lie: smaller the values, less skewed the results.

A reflexive thematic analysis approach was used to analyse the qualitative data [24, 25] since the theoretical freedom of this approach allows flexibility. Following the familiarization with the data through transcription, reading, and re-reading, initial codes were generated and gathered into potential themes. After reviewing themes across an entire data set, a thematic map was developed, and clear definitions and names for each theme were identified and further refined through ongoing analysis. The data were coded by one author (VT) with an inductive approach and themes were developed at a semantic level. Concepts of data or code saturation were not used in this study because they were not consistent with reflexive thematic analysis values and assumptions [24]. Taxonomy was developed manually and independently at the same time by another researcher (MMK), and the taxonomy choice finalized by the third researcher (AM). Minimal grammar and spelling corrections were made to participants’ answers by a researcher that is a native English speaker (MEE). In round 2 of the DS, a taxonomy based on the responses from the first surveys was presented to participants.

Data gathered with the online survey system were exported to Microsoft Excel and SPSS 16 + compatible files and then analysed with NVivo 12 Plus for Windows (QSR International Pty Ltd., London, UK), Microsoft Excel 2013 (Microsoft Corporation, Redmond, WA, USA) and IBM SPSS Statistics for Windows, version 28.0 (IBM Corp., Armonk, NY, USA).

## Results

A total of 55 participants were invited and 39 agreed to participate. In round 1, 33 completed the survey in its entirety, 3 participants opted out, and 3 participants did not provide complete responses. These 33 participants were invited to the round 2, and 24 of them completed it. Since the consensuses were reached after sending 3 reminders 2 weeks apart from each other, the survey was ended.

### Participants’ characteristics

**Fig. 1:**
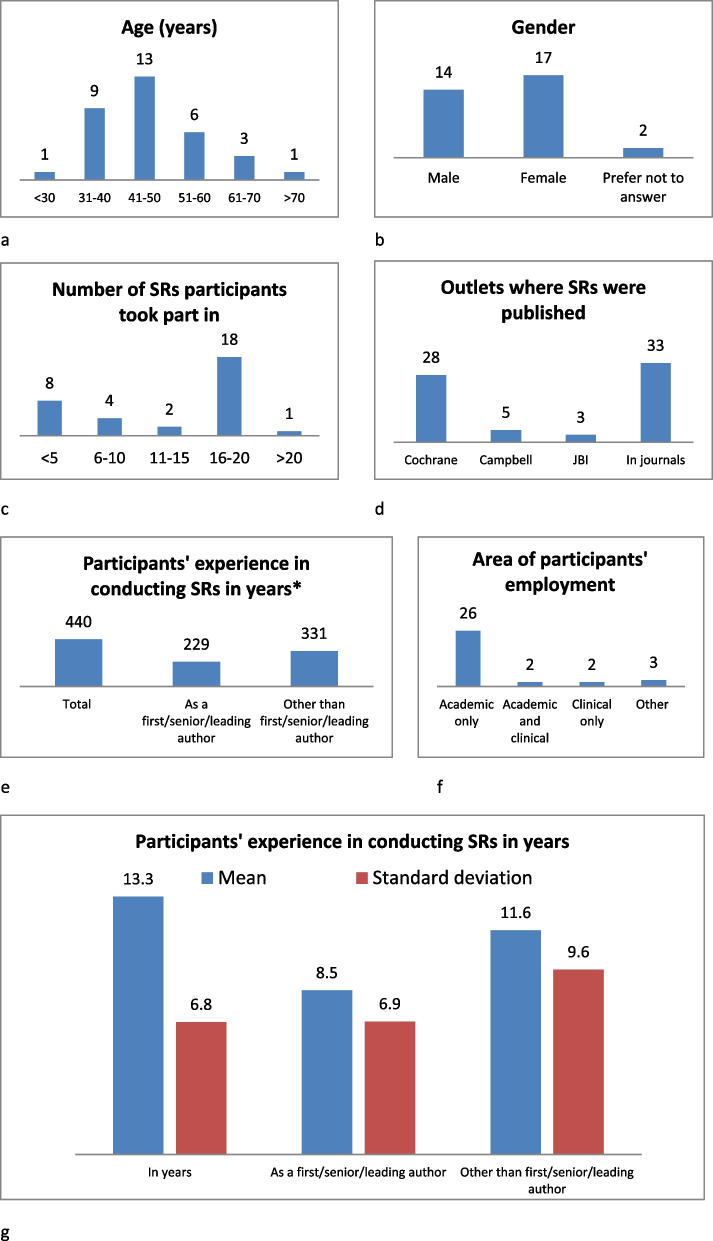
Demographic characteristics of participants. Since there were no extreme outlier values, mean and standard deviation measures were selected as central tendency and level of dispersion measures.*if respondent's answer was “more than *x*”, “around *x*” etc. the value was calculated as *x*

Of the 33 participants who completed the questionnaire in round 1; 14 identified as male, 17 as female, and 2 of them preferred not to specify their gender. There were representatives of all age groups, but most participants were aged 41–50 years (*n* = 13), or 31–40 years old (*n* = 9). Since the average experience in conducting SRs was 13.33 years, it can be stated that our participants were experts in the field (Fig. 1).

### Descriptive data analysis

**Fig. 2:**
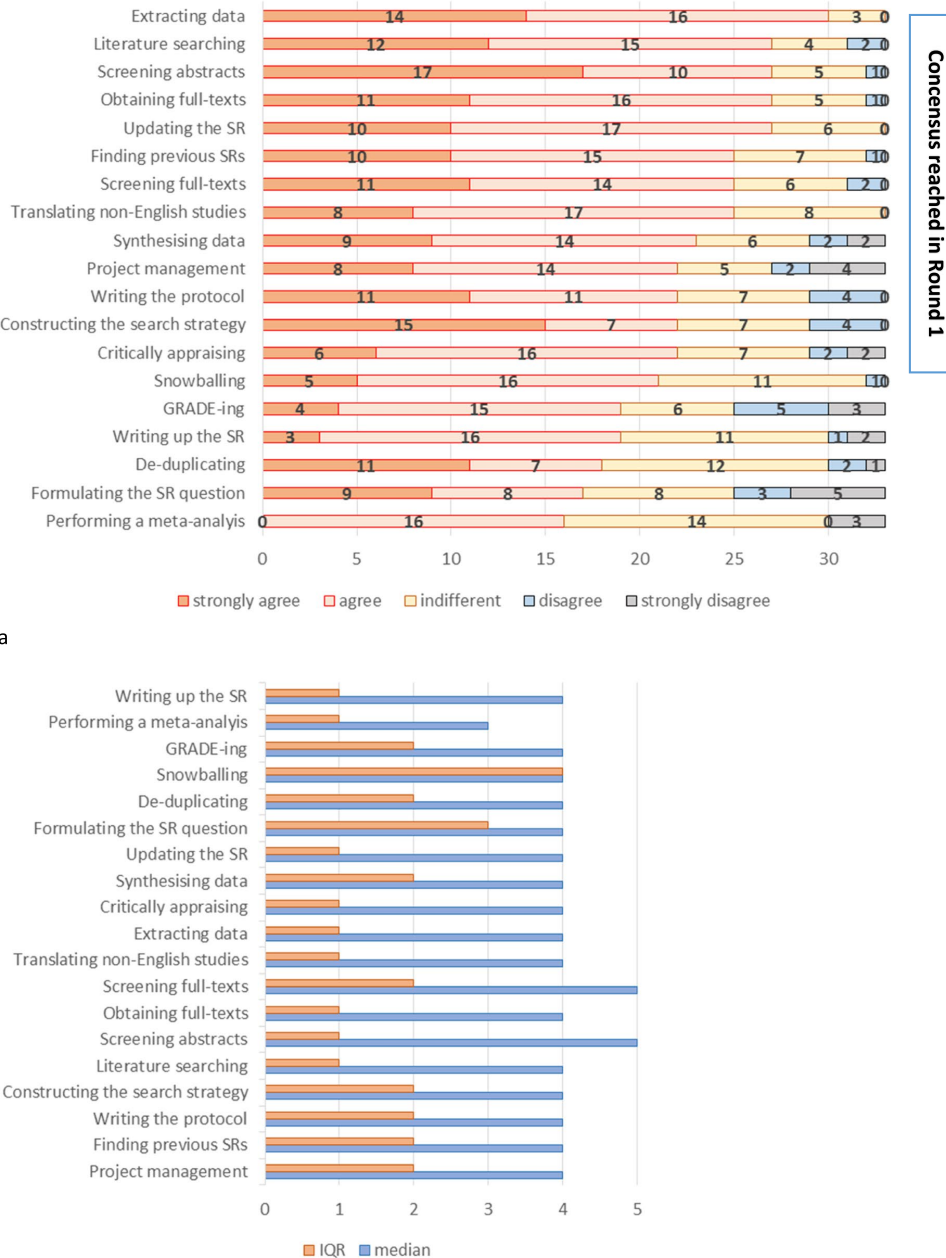
Results of round 1 of the DS. Participants’ view on to what degree the step of the SR production and updating should be prioritizied concerning methods of development and automation

For the general Likert-scale questions in round 1, with regards to identifying which areas and methods of the process of conducting SR are most time/effort/resource-intensive and should be prioritized in future research concerning methods of development and automation, consensus was reached (more than 66.7% participants agreed or strongly agreed that topic should be prioritized) for 13 out of the 19 topics, as is shown in Fig. 2. Data extraction was prioritized by the majority as an area where research and automation can help reduce the intensity of resource use; 90% (30 out of 33) have “strongly agreed” or “agreed”; which was also emphasized in qualitative results section. Screening abstracts has the most “strongly agree” answers 51% (17 out of 33) and by adding “agree” it comes third 82% (27 out of 33). Great majority of participants (27 out of 33; 82%) prioritized literature searching (12 participants “strongly agree” and 15 “agree”), obtaining full texts (11 participants “strongly agree” and 16 “agree”) and updating the SR (10 “strongly agree” and 17 “agree”).

**Fig. 3:**
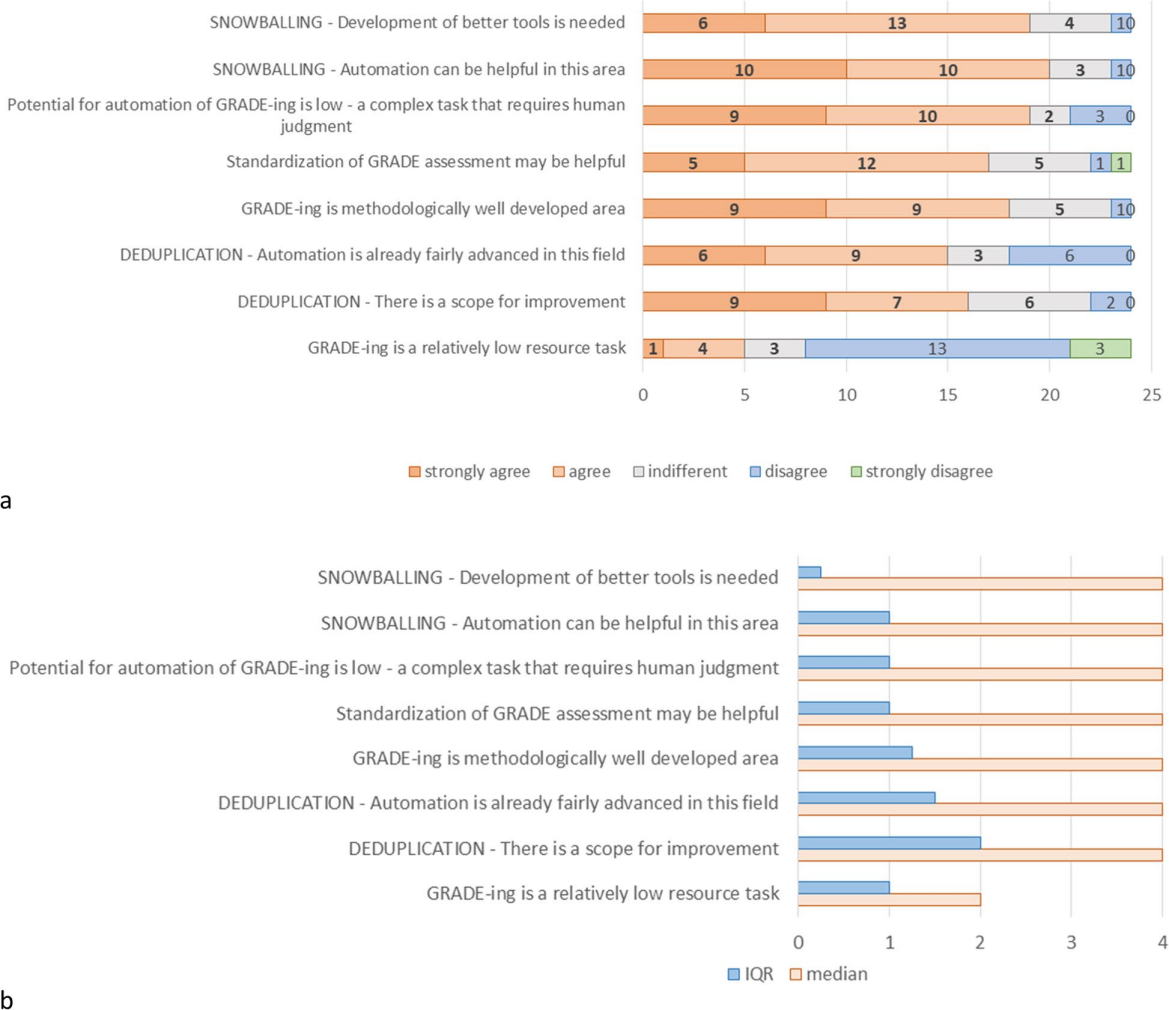
Results of round 2 of the DS. Participants’ view on snowballing (development on better tools, need for automation), GRADE-ing (complex task that requires human judgement, standardization, resource use, methodologically developed area), and deduplication (scope for improvement, advancement of automation)

As depicted in Fig. 3, during round 2, participants reached a consensus on all 8 tested statements with 66.7% either agreeing/strongly agreeing or disagreeing/strongly disagreeing. The most supported statements from round 2 are for snowballing and GRADE-ing: 83% or 20 out of 24 participants reached consensus (10 “strongly agree”, 10 “agree”) that automation can be helpful in snowballing; 79% or 19 out of 24 reached consensuses (9 “strongly agree”, 10 “agree”) that GRADE-ing is a complex task that requires human judgement and potential for automation is low in this activity. The latter consensus of 79% was also reached regarding statements that GRADE-ing is methodologically well developed (9 “strongly agree” and 9 “agree”), as well as development of better tools in snowballing (6 “strongly agree” and 13 “agree).

### Qualitative data analysis

**Fig. 4:**
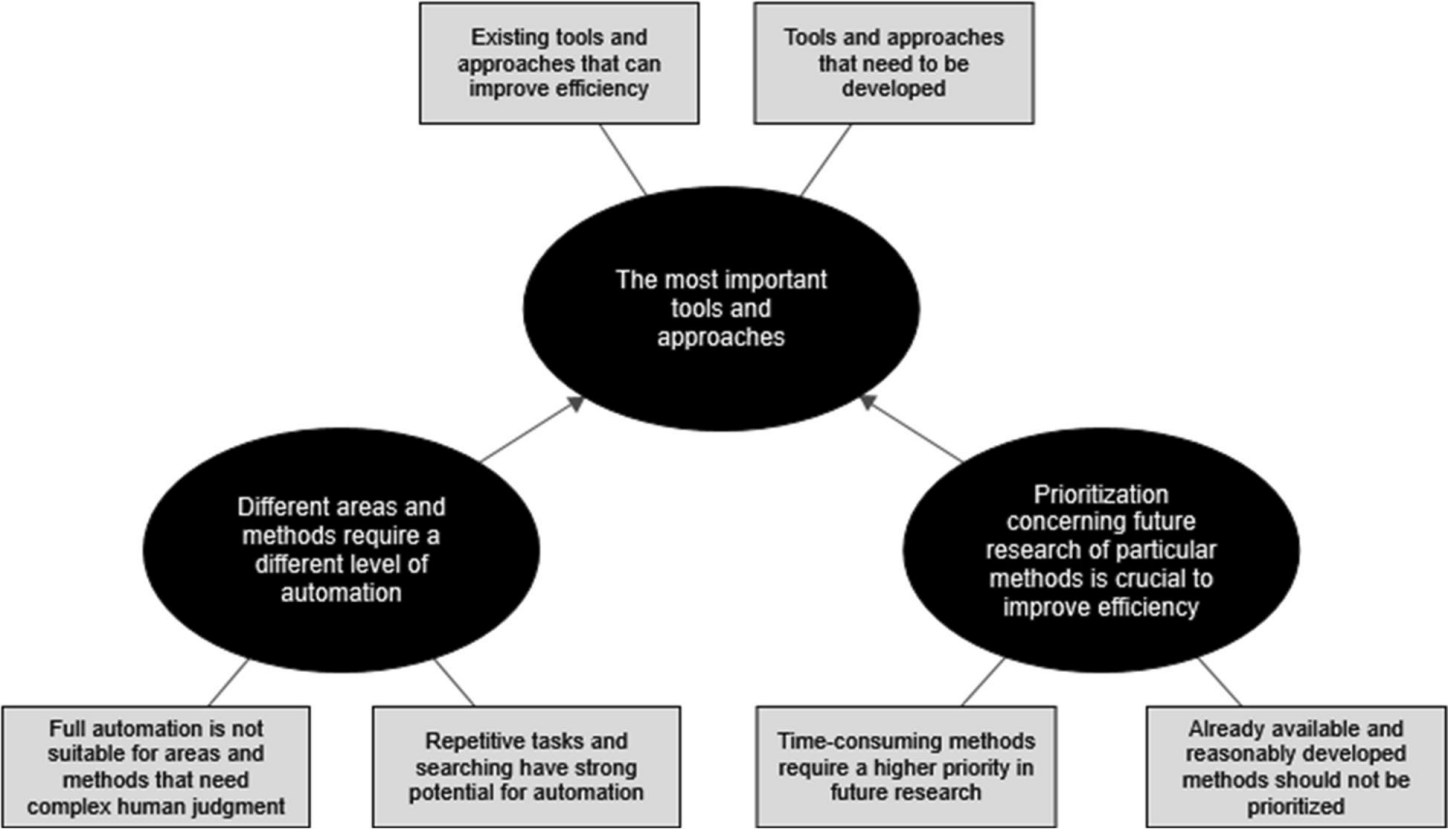
The most promising areas and methods to improve the efficient production of SRs thematic map. The reflexive thematic analysis of qualitative data identified 3 main topics and 6 subtopics

The following three main themes were developed from the qualitative data gathered in the DS: (1) the most important tools and approaches, (2) different areas and methods require different levels of automation, and (3) prioritization concerning future research of particular methods is crucial to improve efficiency (Fig. 4).

The first theme (Table 2) developed in our qualitative study is regarding what are considered the most important tools and approaches used to produce and update SRs. Participants mostly pointed out tools and approaches that are already available on the market but are often not known or used by most researchers. However, they also recommended some tools and approaches that need to be developed to improve the efficiency of SRs. As a result, two sub-themes were developed: “Existing tools and approaches that can improve efficiency” and “Tools and approaches that need to be developed”.

**Table 2:** Qualitative analysis—themes, subthemes, and participants’ statements

Theme	Subthemes	Participants’ statements
The most important tools and methodological approaches	Existing tools and approaches that can improve efficiency	*P9: Already methods (SUAMRI, What review is right for you, IEBHC Review Wizard)* *P2: Revman and most other organizations give templates to fill in details* *P16: […] but there are a number of excellent tools being developed for this at the Institute for Evidence-Based Healthcare at Bond University in Australia—*https://iebh.bond.edu.au/education-services/research-tools *P16: I know there are tools for this [De-duplicating], either standalone (e.g., Institute for Evidence-Based Practice at Bond University) or integrated into systematic review software—Covidence, EPPI-Reviewer* *P11: [Screening full-texts] Already explored in some commercial platforms (i.e., Distiller, EPPI-Reviewer)* *P16: This [Screening full-texts] is harder to automate at the moment, and is quite a manual task, so a productive area for research* *P7: Using a program like Covidence or DistillerSR or EPPI-Reviewer to screen the search, perform a risk of bias, extract data—such a program will make sure that all parts of the process are accounted for* *P32: this [Snowballing] is important but great tools like Citation chaser already exist* *P7: Use Google Translate whenever there is a non-English publication—Google Translate only trustworthy from any language to English* *P11: Several platforms for translation are already available (i.e., DeepL)* *P16: Google Translate as a tool has increased dramatically in quality over the years, and guidance now recommends it can be used for study screening, if not final full-text translation for incorporation into the review* *P14: Prioritisation of teaching how to use systematic review automation tools in training (e.g., JBI, Cochrane, other organisations)* *P21: Regular and in-depth training is important* *P14: Prioritisation of teaching how to use systematic review automation tools in training (e.g., JBI, Cochrane, other organisations)*
	Tools and approaches that need to be developed	*P2: Finding previous systematic reviews is straightforward (provided they are published or registered) hence I believe not much development and automation is required. However, if there is a platform wherein all the registered and published reviews are pulled together like a repository it may benefit researchers* *P16: Repositories for sharing translations might also be useful, reducing resource use through duplication of effort* *P16: I have also heard discussions about possible platforms for sharing extracted data across reviews, which could reduce duplication* *P2: Also, rather than searching in various databases separately, each database can be linked to having one common search strategy, which I believe will save a lot of time* *P32: One of the most time-consuming parts of a review is wading through irrelevant records made necessary by inconsistent indexing in databases and different syntax in different databases. Findings a way to run a search in one place that would automatically search all sources would be wonderful* *P8: Machine learning is helping but even more could be done* *P16: [Screening full-texts] Tools which make this easier, including summarising the results/reasons (e.g., Covidence, EPPI-Reviewer) are already useful* *P27: This [Screening abstracts] is very tedious and time-consuming task. Artificial intelligence or citizen science might help with that* *P24: [Extracting data] More challenging to automate, but high value if it can be made to work* *P13: I think that there are many nice tools already. But two areas which are very time consuming and would benefit from new tools (in my opinion) are finding full text and extracting data* *P2: Many researchers have to restrict the search to English languages because of resource constraints. If this area is developed, it will be very good* *P12: this [Translating non-English studies] would be a game changer….If…it went beyond quantitative data, and qualitative researchers could be confident the 'meaning' of text was captured*
Different areas and methods require a different level of automation	General	*P24: I think the potential for automation to help is limited here [Project management]* *P11: I am not sure if this step [Formulating the review question] could be automated* *P27: Anything related to search obviously has a potential for automation, therefore worth researching* *P12: [Screening abstracts] potentially the strongest area of contribution for smart automation*
	Full automation is not suitable for areas and methods that need complex human judgment	*P32: The most time-consuming parts of the process are those that lend themselves best to some degree of automation—deduplication, screening, data extraction. The other parts, I feel, require expert human input especially where complex decisions need to be made* *P22: […] the systematic review process is a multi-step process, nearly all of which require judgments. And you cannot automate judgments, and perhaps should not, even in the face of artificial intelligence (AI)* *P6: [Formulating the review question] this is a piece of the endeavour that I can't see being done for a machine. Whether it is theoretically possible to find ways around this limitation, I doubt research on automation in this area will provide many things* *P5: [Writing the protocol] Some parts of the protocol follow rules and standards, that can be supported by software and text template, while other elements (e.g. study selection criteria, inclusion of non-randomized evidence) are complex questions, which have to be left to human minds* *P5: [Writing up the review manuscript] Automatic links between text and key statistical findings are useful, but writing the introduction and discussion section requires a human mind* *P32: [Constructing the search strategy] I don't know that automation will improve things, a more pressing issue is SRs conducted without input from an info specialist* *P7: [Literature searching] Difficult to automate, always needs a human to make decisions, but crucial for a systematic review* *P7: [Screening full-texts] I am afraid that human decisions are needed* *P32: I am sceptical on automation here [Screening full-texts] especially in the social sciences where there is so much inconsistency on how studies are reported that it takes a human to find the relevant information* *P7: [GRADE-ing] This process is dependent upon judgements that I can´t imagine a meaningful automation or semi-automation* *P8: [GRADE-ing] I do not think that GRADE decisions should be automatic as they require a lot of reflection and thought. There are already existing tools that are functional* *P16: [Synthesizing data] I think this is a stage of the review where it's really productive and important for authors to spend time and energy to do this thoughtfully and appropriately—at this stage I think there are risks in trying to automate this, as judgement is needed before proceeding to synthesise data* *P32: [Synthesizing data] with more complex data sets having a human checking statistics and conversions of different measures to a common effect estimate takes real skill and knowledge of stats so I am sceptical about automating that process* *P7: [Extracting data] My experience is that it is very demanding and includes a lot of decisions by humans, but a combination would be good* *P3: [Extracting data] Semi automation perhaps?*
	Repetitive tasks and searching have strong potential for automation	*P4: Researchers need to understand that automation should prevent them from having to do rote, complicated, repetitive tasks—thus freeing them up to do more interesting and critical tasks. I.e. automation is a tool for them to have more of a difference, whether in evidence-based medicine or policy. It is not a replacement for them* *P17: Searching and analysing relevance are most likely places for automatization, perhaps also the data extraction* *P23: AI helps reducing the same work* *P27: Anything related to search obviously has a potential for automation, therefore worth researching. Especially in the area in qualitative evidence synthesis we still have a lot to research and learn*
Prioritization concerning future research of particular methods is crucial to improve efficiency	General	*P13: I think that there are many nice tools already. But two areas which are very time consuming and would benefit from new tools (in my opinion) are finding full text and extracting data* *P3: [De-duplicating] Less important, as already fairly advanced* *P27: [De-duplicating] Isn't that automated already?*
	Time-consuming methods require a higher priority in future research	*P32: [Literature searching] One of the most time-consuming parts of a review is wading through irrelevant records made necessary by inconsistent indexing in databases and different syntax in different databases. Findings a way to run a search in one place that would automatically search all sources would be wonderful* *P2: [Obtaining full-text] This step takes a lot of time and thus to save time prioritization in future research in needed* *P13: [Extracting data] This is the most time consuming part where to my knowledge there are no good tool available* *P16: [Extracting data] This is a very time consuming process and existing tools can be challenging to use, especially for complex reviews with multi-component, highly variable interventions and a lot of variability in how outcomes are measured and reported. Ongoing improvement of data extraction tools would be great, as would semi-automation to assist in identifying and classifying relevant information and reduce author workload. I have also heard discussions about possible platforms for sharing extracted data across reviews, which could reduce duplication*
	Already available and reasonably developed methods	*P32: [Writing the protocol] Guidance on protocols is widely available and experienced reviewers can write a quality protocol swiftly* *P11: [Obtaining full-text] Already available in most of the commercial software for management of references* *P6: Whether that [Translating non-English studies] could be extremely valuable in reviews, a lot of research and experimenting is already been done in this area, so prioritizing it in the context of reviews seems unlikely to produce additional benefits*
Open-ended comments	General	*P10: “We should develop methods to combine different study designs and generate evidence.”* *P10: “Non-English databases are usually not included in the systematic reviews. For example, it is difficult to get access or translation facilities for Chinese databases. Thereby we are missing a huge chunk of information that could have an impact on the results of the systematic review.”* *P24: “You cannot automate judgements, and perhaps should not, even in the face of artificial intelligence (AI)” “In a bit more ‘intellectual’ activities like RoB, synthesis and GRADE-ing I am sceptic towards the automatization” “I think one of the challenges when thinking about automation is that people tend to think (as in the case in the survey) of automation is that people tend to think (as in the case in the survey) of automation supplement/replacing/assisting in existing human processes.”* *P22: “The theoretical framework regarding the SR methodology is clear and valid. Available research shows inconsistent judgements on the risk of bias, methods around data synthesis that are not always appropriate, and we may question the assessment of certainty of evidence…We should start with this; identifying areas of SR methodology that have shown to be inconsistent.”*

For areas such as “Formulating the review question” and “Writing the protocol”, the participants recommended SUAMRI, IEBHC Review Wizard, and RevMan. They also pointed out several other tools developed by the Institute for Evidence-Based Healthcare at Bond University in Australia: Systematic Review Accelerator (SRA), PRISMA for Abstracts, TIDieR, Shared decision making, EBM teaching resources, CriSTAL Tool, and MASCoT.

Regarding de-duplicating, screening abstracts, and full-texts, the expert suggested that tools such as EPPI-Reviewer, Covidence, DistillerSR can improve the efficiency of SRs.

Some participants pointed out that some handy tools already exist, such as “Citation chaser” (https://estech.shinyapps.io/citationchaser/) that can be used for citation tracking.

The participants also pointed out that the quality of available tools for translating non-English studies has increased dramatically over the years, suggesting that Google Translate and DeepL seem to be the most useful tools in this area.

Additional training on how to use tools and training in general was seen as something that could improve the overall quality of SRs.

The participants suggested various tools and approaches that need to be developed to increase the efficiency of SRs. Some participants suggested the need for repositories for all registered and published reviews that can be used to find previous SRs and share translations or extracted data across reviews.

One possible approach to improving SRs’ efficacy would be a common search strategy for all databases. Automation of screening abstracts and full texts is considered one of the most needed tools in SRs. Development of tools for automation in extracting data could be highly beneficial, so future research in that area is worthwhile. Some of the participants also mentioned the need for further development of machine learning to improve the efficiency of SRs, especially for finding full texts.

Development of more advanced tools for the translation of non-English studies was proposed by few participants.

Automation was one of the most frequently mentioned topics in round 1 of our DS. Some participants suggested that particular areas and methods of SRs have a limited potential for automation, and that some parts of the SR process are impossible to automate.

On the other hand, some participants mentioned areas and methods with a strong potential for automation. The components most frequently mentioned with a strong potential for automation are searching and screening.

Since the participants most frequently described some areas and methods as more suitable for automation than others, two additional sub-themes were also developed: “Full automation is not suitable for areas and methods that need complex human judgment” and “Repetitive tasks and searching have strong potential for automation”.

Some participants believed that the components of SR that require complex human judgments are not suitable for automation. One participant emphasized that automation of judgments should not be favourable even if artificial intelligence would allow it.

The participants mostly viewed areas and methods connected with writing as less suitable for automation, specifically the writing up the protocol or manuscript and formulating the review question.

Although some experts considered activities connected with searching as most promising for automation, others suggested that humans still need to make decisions in those areas.

Screening full texts was specified as a part of searching that is not suitable for automation, especially in social sciences where inconsistency on how studies are reported is common.

GRADE-ing or going from evidence to decision is another process that was seen as dependent upon human judgements.

The participants also pointed out that synthesizing data is a complex process, which is why they remain sceptical about its automation.

However, other participants suggested that some areas and methods that need complex human judgments could benefit from semi-automation in which a combination of automation and human judgment will be utilized in the decision-making process.

The participants discussed areas and methods in which automation could be worthwhile. They concluded that repetitive tasks and activities connected with searching have the most substantial potential for automation.

Some participants mentioned in their comments that some areas are very time-consuming, so additional research and development of new tools would be beneficial for improving the efficiency of SR.

Others pointed out that some areas and methods are already developed or automated, which is why they are less important for future research.

Therefore, a third theme emerged: prioritization concerning future research of particular methods to improve the efficiency of SRs in general. Two sub-themes were developed within this theme: “Time-consuming methods require a higher priority in future research” and “Already available and reasonably developed methods should not be prioritized”.

Most participants believed that the SR process stands to benefit the most from future research aimed at improving the time-consuming components.

Extracting data was seen as one of the most time-consuming parts of SR, so the participants especially emphasised prioritising that area in future research.

The participants mentioned several methods that are reasonably developed and already available on the market.

They emphasized that already existing methods and areas should not be prioritized in future research since it seems unlikely to produce additional benefits for SR efficiency.

One participant mentioned that there is a need to increase the integration of different study designs into producing evidence.

Although many participants stated that there are good enough translating tools available, the language barrier still resembles a huge obstacle in SRs’ production.

Many participants agreed that there is a lot of automation achieved in the area of SRs’ production and updating, but there are still tasks that cannot and should not automatized since they rely on complex human judgements.

Several participants emphasized that methodology of SRs’ production is fairly advanced.

## Discussion

The main finding of this DS was that extracting data, literature searching, and screening abstracts as the most important areas to be prioritized in future research when developing SR methods and tools.

There is a consensus among participants that “snowballing” is a relatively low resource task, development of better tools is needed, and automation can be helpful in this area. One participant (P32) mentioned an efficient tool that has a great potential to successfully automate this step: *Citationchaser*. There are some software solutions already available that support basic forms of snowballing/citation chasing. *Citationchaser* [26] is an open source, easy-to-use tool for quick backward and forward citation chasing, developed by Haddaway et al. [27], and seems to be the most advanced in the field. It generates standardized output files that can be quickly and effectively combined with the results of bibliographic database searches to reduce duplication and increase the breadth of the pool of potentially pertinent records that can be screened within an evidence synthesis [27]. The fact that only one participant was aware of existing of this tool, among our highly expert panel, grants that this tool has to be further developed and popularized.

Participants also agreed that potential for automation of “GRADE-ing” is low since this is a complex task that requires human judgement, and that methodologically this is a very well-developed area and standardization of GRADE assessments may be helpful. Regarding “deduplication,” experts agreed that automation is already fairly advanced, but there is room for improvement.

Regarding possible area of improvements in methodology, several participants emphasized that automation is not a panacea and has to be used to “prevent from having to do rote, complicated repetitive tasks”, “automation is a tool…to have more difference…it’s is not a replacement for human judgement”.

Including non-English studies in SRs has been recognized as important to avoid bias, although reviewers commonly report that it is costly and time-consuming to include them, and previously have been reluctant to bother with the language barriers [28]. “Many researchers have to restrict the search to English language because of resource constraints”. Participants from our DS showed awareness and willingness to incorporate non-English evidence in their SRs. In their comments, participants emphasized the importance of this issue, especially in the qualitative area: “This would be a game changer…qualitative researchers could be confident the ‘meaning’ of text was captured”. Many participants emphasized that quality of available language translating tools has dramatically increased over the years, specifically pointing out Google translate and Deep L, which is promising, and hopefully will progress into qualitative filed of research in the future.

### Limitations

Limitations of the study stem from the very nature of the research method: one can always debate that there were additional “expert” SRs’ authors who could have better answered the survey. Efforts were made to select experts who were relatively impartial yet had interest in the research topic and were willing to spare their precious time. The EVBRES collective knowledge of the SRs’ production landscape was excellent base for handpicking the best available sample and serve as effective gatekeepers [18]. In fact, participants demonstrated vast experience (totalling 440 years in conducting SRs) at a relatively young age (most panellists were 41–50 years of age) (Fig. 1). Most of the participants (26/33) work in academia: it is highly understandable that researchers from that area are the most efficient producers of SRs. Participants published SRs in various settings: all (*n* = 33) have published in various journals, and the majority (*n* = 28) published Cochrane SRs. Another limitation is also due to the nature of DSs: the Delphi method has been criticized in that it does not allow participants to discuss the issues raised and gives no opportunities for participants to elaborate on their views, resulting in the potential risk that greater reliance is placed on the results than might be warranted [18]. DSs also have additional limitations, such as not allowing the same level of interaction or fast turnaround that is possible, for example, in a focus group. However, this also presents a strength due to the fact that participants do not meet with each other face to face, and therefore they can present and react to ideas unbiased by the identities and pressures of others [29].

## Conclusions

The participants recommended tools and approaches that can improve the efficiency of SRs. Data extraction was prioritized by the majority of participants as an area that needs more research/methods development, where research and automation can help reduce the intensity of resource use. They specified that some areas and methods are more suitable for automation than others, e.g., snowballing, and development of tools is needed in this area. There is an open-source tool—Citation chaser, which has a high potential to present a significant time saving in the SRs production process. GRADE-ing was identified as an area that is methodologically well developed, a complex task that has lowest potential for automation, as it requires high level of human judgement.

As expected, s of SR automation is already developed and less critical for future research (GRADE-ing), om additional research and the development of new tools.

## Data Availability

Anonymized datasets used and/or analyzed during the current study are available from the corresponding author on reasonable request.

## Abbreviations

SR: Systematic review
DS: Delphi study
JBI: Joanna Briggs Institute
WG: Working group
COST: European Cooperation in Science and Technology
EVBRES: EVidence-Based RESearch
